# Prevalence of *pks*-positive *Escherichia coli* in Japanese patients with or without colorectal cancer

**DOI:** 10.1186/s13099-017-0185-x

**Published:** 2017-06-12

**Authors:** Takayuki Shimpoh, Yoshihiro Hirata, Sozaburo Ihara, Nobumi Suzuki, Hiroto Kinoshita, Yoku Hayakawa, Yumiko Ota, Akiko Narita, Shuntaro Yoshida, Atsuo Yamada, Kazuhiko Koike

**Affiliations:** 1 Department of Gastroenterology, Graduate School of Medicine, The University of Tokyo, 7-3-1 Hongo, Bunkyo-ku, Tokyo, 113-8655 Japan; 2 Department of Gastroenterology, The Institute for Adult Diseases, Asahi Life Foundation, Tokyo, Japan; 3 Department of Endoscopy and Endoscopic Surgery, The University of Tokyo Hospital, Tokyo, Japan

**Keywords:** Colibactin, Colonoscopy, Colorectal neoplasms, *Escherichia coli*, *pks* island

## Abstract

**Background:**

Recent studies show that some *Escherichia coli* strains possessing a gene cluster named the *pks* island might have a causative role in the development of human colorectal cancer (CRC). In several reports from Europe, they are found more prevalently in colon tissue specimens derived from CRC patients compared to those from controls. In this study we sought to clarify the difference in *pks* prevalence between CRC patients and non-CRC controls in the Japanese population, by using non-invasive sample collection technique during colonoscopy.

**Methods:**

Colonic lavage samples were collected during diagnostic colonoscopy, and bacterial DNA within each sample was extracted. Fecal DNA samples were then examined for *pks* island genes using conventional qualitative PCR and real-time quantitative PCR. In some patients biopsy samples were also collected in the same session of colonoscopy, and the correlation between the *pks* status of the colonic lavage sample and the biopsy sample of the same patients was evaluated.

**Results:**

Twelve out of thirteen patients (92%) showed the same *pks* status by colonic lavage sample and biopsy sample, suggesting the usefulness of colonic lavage samples as a surrogate for biopsy samples. A total of 98 colonic lavage samples were collected, which included 35 from CRC patients, 37 from adenoma patients, and 26 from controls. The *pks*-positive bacterial DNA was detected in 43, 51, and 46% of colonic lavage samples from CRC, adenoma, and control patients, respectively, and there was no significant difference among diseases. Real-time quantitative PCR showed no significant difference in the relative concentrations of *pks*-positive bacterial DNA among diseases. Age, gender, location of CRC, CRC staging, or *k*-*ras* gene status was not associated with *pks* prevalence.

**Conclusions:**

Although the method of collecting fecal DNA from colonic lavage samples was safe and technically feasible, factors other than *pks*-positive bacteria appear to play more important roles in CRC development in this cohort.

## Background

Infectious microorganisms are often thought to be associated with human carcinogenesis. For example, *Helicobacter pylori*, a Gram-negative bacterium chronically residing in the human stomach, is referred to as a definite carcinogen of gastric cancer [1–3]. Human hepatitis B and C viruses are also established as causative agents of hepatocellular carcinoma [4, 5]. Similar association is also established between some human papilloma viruses and carcinoma of the cervix uteri [6].

Colorectal cancer (CRC) is the fourth leading cause of cancer-related deaths in the world [7], and its prevention and early detection are among the most urgent needs regarding public health. Human colon is colonized by more than 10^14^ bacteria which can be classified into at least 1000 species, making up what is called the microbiota, and it would be natural to assume that some of these bacteria are associated with the pathogenesis of human CRC. Recently, several studies have indicated that certain strains of *Escherichia coli* (*E. coli*) possessing a gene cluster named the *pks* island might have a causative role in human CRC development [8, 9]. *E. coli*, a Gram-negative, facultative anaerobic rod, a member of the family *Enterobacteriaceae*, is found widely in the gastrointestinal tract of many mammals, including almost all humans. This species is further divided into phylogenetic groups A, B1, B2, and D. Most strains belonging to group B2 are known for their extraintestinal pathogenic nature causing urinary tract infection, sepsis, and newborn meningitis in humans, which are called extraintestinal pathogenic *E. coli* (ExPEC), whereas strains in groups A and D are mostly nonpathogenic commensals or pathogens which mainly cause intestinal disorders presenting as diarrhea [10, 11]. The *pks* island, made up of approximately 54,000 base pairs, consists of genes coding three nonribosomal peptide megasynthases (NRPS), three polyketide megasynthases (PKS), and two hybrid NRPS/PKS megasynthases [12], and is thought to produce a peptide–polyketide genotoxin named colibactin. This pathogenic island is found mostly in phylogenetic group B2 *E. coli* [9, 13, 14], although there are also a much smaller number of *pks*-positive strains in group B1 *E. coli* and other bacterial species in the family *Enterobacteriaceae* [15].

Previous in vitro experiments showed that *pks*-positive *E. coli* induces DNA double-strand breaks and transient G2-M cell cycle arrest in its host mammalian cells, whereas *pks*-negative *E. coli* does not [13, 16]. Those infected host cells can survive after incomplete DNA repair, resulting in higher mutation rates, presumably leading to tumorigenesis. They also showed that *pks*-positive *E. coli* loses this genotoxic property by knocking out the *pks* island, while *pks*-negative *E. coli* acquires genotoxicity by introducing a gene construct containing the *pks* island [13, 16]. In vivo studies used colitis-susceptible interleukin-10-deficient mice under germ-free condition, and when they were associated with either *pks*-positive *E. coli* or *pks*-negative *E. coli*, together with azoxymethane, the group of mice receiving *pks*-positive *E. coli* showed significantly greater increase in colon tumor occurrence and invasiveness compared to the other group, while the severity of colitis was similar between the two groups [8]. When host cells are infected with greater number of *pks*-positive *E. coli*, they show senescence-associated secretory phenotype, by which the cells secrete growth factors such as hepatocyte growth factor, stimulating non-infected neighboring cells to proliferate, also possibly causing tumor formation [17].

Concerning humans, two studies from Europe showed that *pks*-positive *E. coli* are more prevalent in colon tissue specimens derived from CRC patients compared to those from non-CRC controls. Arthur et al. studied on tissue specimens from 21 CRC patients and 24 controls and found *pks*-positive *E. coli* from 67 and 21% of each category, respectively [8]. Likewise, Buc et al. studied on tissue specimens from 38 CRC patients and 31 diverticulosis patients and found *pks*-positive *E. coli* from 55 and 19%, respectively [9]. Thus, the *pks* island may act as a tumor promoter for CRC, and could be used as a predictive biomarker for CRC development.

In the present study, we sought to clarify the difference in *pks* prevalence between CRC patients and non-CRC controls in the Japanese population, by using non-invasive endoscopic sample collection technique.

## Methods

### Patients

Patients who received total colonoscopy at the University of Tokyo Hospital from January 2014 through May 2015 were asked for participation, and written consent was obtained from every patient who agreed to participate. Clinical characteristics and history of previous and present endoscopic findings of each patient were recorded. Patients were classified into three disease categories: CRC, adenoma, and control; regardless of whether the disease was diagnosed by previous or present session of colonoscopy. In all CRC patients, endoscopic diagnosis was confirmed by pathological examination of previous or concomitant biopsy samples. *k*-*ras* gene status of the tumor was determined by direct sequencing of tumor DNA extracted from paraffin-embedded tumor tissues.

This study was approved by the ethics committee of the University of Tokyo (approval number 10329).

### DNA extraction from colonic lavage samples and biopsy samples

Ten milliliters of residual suspension in the colonic lumen after lavage was collected during colonoscopy of every patient, using a syringe connected to the endoscope. The samples were then transiently stored and transported at 4 °C. Solid component within each suspension sample, which contained bacterial cells, was isolated by centrifugation and was washed with phosphate buffer saline for three times, in order to remove possible water-soluble distractors such as polyethylene glycol contained in oral bowel preparation solution. Then total bacterial DNA was extracted using QIAamp DNA Stool Mini Kit (QIAGEN), and was stored at −30 °C until use.

Biopsy samples were washed with phosphate buffer saline in order to remove mucus and mucus-associated bacteria. DNA was extracted from the tissue and co-existing mucosa-associated bacteria using QIAamp DNA Mini Kit (QIAGEN), and was stored at −30 °C until use.

### PCR analysis

Both conventional qualitative PCR and real-time quantitative PCR were used to evaluate fecal DNA samples. In order to detect *pks*-positive bacterial DNA, primers were used to amplify a 283 bp sequence in *clbB* gene of *E. coli*, a part of the *pks* island, with the forward primer 5′-GCGCATCCTCAAGAGTAAATA-3′ and the reverse primer 5′-GCGCTCTATGCTCATCAACC-3′ [8]. Primers to amplify a 147 bp sequence in *E. coli*-specific gene *uidA* with the forward primer 5′-TGGTAATTACCGACGAAAACGGC-3′ and the reverse primer 5′-ACGCGTGGTTACAGTCTTGCG-3′, and those to amplify a 144 bp universal bacterial sequence in 16S rRNA gene with the forward primer 5′-GGTGAATACGTTCCCGG-3′ and the reverse primer 5′-TACGGCTACCTTGTTACGACTT-3′, were also used in order to detect total *E. coli* DNA and total bacterial DNA, respectively [18, 19].

Twenty microliters of reaction mixture for conventional qualitative PCR consisted of 1 μL of template DNA solution, each 1 μL of 10 μmol/L forward and reverse primer solutions, deoxynucleotide mixture, the DNA polymerase AmpliTaq Gold (Applied Biosystems), and buffer according to the manufacturer’s instructions. The PCR conditions were 7 min at 95 °C, 35 cycles of 30 s at 95 °C, 30 s at 55 °C, and 30 s at 72 °C, and 7 min at 72 °C.

Patients were considered *pks*-positive when their fecal bacterial DNA tested *pks*-positive after conventional qualitative PCR, and for each disease category, *pks* prevalence was defined as the proportion of the number of *pks*-positive patients to that of total patients in that category.

We have isolated several *E. coli* strains from patients. One of these isolates was positive for *clbB* gene, *uidA* gene, and 16S rRNA gene by conventional qualitative PCR and DNA sequencing. This isolate was also positive for *clbQ* and *clbA* genes, which are located near the 5′ and 3′ terminals of the *pks* island, respectively, thus suggesting this isolate contained the entire sequence of the *pks* island [14]. Primers to amplify a 308 bp sequence in *clbQ* gene with the forward primer 5′-GCACGATCGGACAGGTTAAT-3′ and the reverse primer 5′-TAGTCTCGGAGGGATCATGG-3′, and those to amplify a 342 bp sequence in *clbA* gene with the forward primer 5′-AAGCCGTATCCTGCTCAAAA-3′ and the reverse primer 5′-GCTTCTTTGAGCGTCCACAT-3′, were used. We used this *E. coli* isolate as the positive control for the *pks* island. As the negative control for the *pks* island and the positive control for *uidA* gene and 16S rRNA gene, str. K-12 substr. MG1655 was used.

Twenty microliters of reaction mixture for real-time quantitative PCR consisted of 2 μL of template DNA solution, each 0.6 μL of 10 μmol/L forward and reverse primer solutions, and the FastStart Universal SYBR Green Master (ROX) mix (Roche) which contains the DNA polymerase. The PCR conditions were 10 min at 95 °C, 40 cycles of 15 s at 95 °C and 60 s at 60 °C, followed by an appropriate dissociation phase.

For each fecal bacterial DNA sample, relative concentration of *pks*-positive bacterial DNA was defined as the proportion of calculated concentration of *pks*-positive DNA given by real-time quantitative PCR to that of total bacterial DNA.

### Statistical analysis

Continuous variables were compared using the Mann–Whitney U test, and categorical variables were compared using the χ^2^ test. All tests were two-sided, and a *p* value <0.05 was considered significant.

## Results

### Detection threshold of pks-positive bacterial DNA

**Fig. 1:**
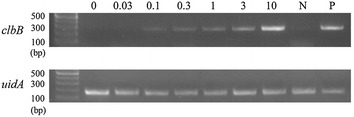
Conventional qualitative PCR to detect various amounts of *pks*-positive control *E. coli* DNA using primers for *clbB* (*top*) and *uidA* (*bottom*) genes. *N* negative control for *clbB* gene (derived from str. K-12 substr. MG1655), *P* positive control for *clbB* gene (referred to in the “Methods” section); numerals, amount (pg/sample) of *pks*-positive *E. coli* DNA. Samples containing 0.1 pg or more of *pks*-positive control DNA tested positive

First we aimed to validate the quality of DNA detection system from colonic lavage samples by using qualitative PCR. We mixed *pks*-positive and -negative *E. coli* DNA together at various ratios, keeping the sum concentration at 500 pg per PCR sample. On conventional qualitative PCR, samples containing 0.1 pg or more of *pks*-positive control DNA tested positive (Fig. 1). Given that *E. coli* genome weighs approximately 5 × 10^−15^ g per copy, this result means that a PCR sample containing DNA from 20 or more *pks*-positive *E. coli*, or a colonic lavage sample (10 mL) containing 1 × 10^4^ or more of them gives a positive result in conventional qualitative PCR.

### Correlation between clbB gene and the whole pks Island

**Fig. 2:**
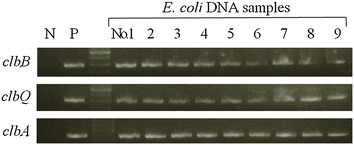
Conventional qualitative PCR to detect *clbB*, *clbQ*, and *clbA* genes from nine DNA samples derived from nine different *E. coli* strains. *N* negative control for *clbB* gene (derived from str. K-12 substr. MG1655), *P* positive control for *clbB* gene (referred to in the “Methods” section)

In order to use *clbB* gene as a surrogate marker of the whole *pks* island, we examined *clbQ* and *clbA* genes, which are located very near to the 5′ and 3′ terminals of the *pks* island, respectively. We selected nine *clbB*-positive DNA samples derived from nine different *E. coli* strains, and found that all of them were also positive for both *clbQ* and *clbA* genes (Fig. 2), which was consistent to a previous study [14]. Therefore, in this study, we used *clbB* gene as a surrogate marker of the whole *pks* island.

### Correlation between colonic lavage samples and biopsy samples

Previous studies aimed to discuss the prevalence of *pks*-positive *E. coli* among mucosa-associated bacteria by using surgical specimens or biopsy samples of the colon. In order to ensure that this study using colonic lavage samples can be compared with those previous studies, we examined the correlation between the *pks* status of the colonic lavage sample and the biopsy sample of the same patients. We obtained a colonic lavage sample and a biopsy sample concurrently during one session of colonoscopy per patient, and collected 13 pairs of colonic lavage samples and biopsy samples from 13 patients. Of those, 12 patients (92%) showed the same *pks* status by colonic lavage sample and biopsy sample (3 patients were both *pks*-positive and the other 9 were both *pks*-negative). One patient provided a *pks*-positive colonic lavage sample and a *pks*-negative biopsy sample (Table 1). Correlation between the *pks* status by the two sampling methods was good, with the κ coefficient 0.806. This result suggested the usefulness of colonic lavage samples as a surrogate for biopsy samples—as far as *pks* status is concerned.

**Table 1:** Correlation between *pks* positivity in colonic lavage samples and biopsy samples

	Biopsy: *pks*-positive	Biopsy: *pks*-negative	Total
Colonic lavage: *pks* positive	3	1	4
Colonic lavage: *pks* negative	0	9	9
Total	3	10	13

### Detection and quantification of the pks-positive *E. coli* by colonic lavage samples

Colonic lavage samples were collected during colonoscopy from 98 patients including 35 from CRC patients, 37 from adenoma patients, and 26 from controls. Each disease category contains past history of respective disease. Gender, age, and amount of total bacterial DNA extracted from each colonic lavage samples are listed in Table 2. Male prevalence was significantly greater among the CRC patients compared to the controls, but otherwise there was no significant difference.

**Table 2:** Patient and sample characteristics, and *pks* prevalence

Disease	CRC	Adenoma	Control	Total
# of patients	35	37	26	98
Age (median, range)	69 (44–97) *p* = 0.223	69 (40–89) *p* = 0.191	66 (37–87)	68 (37–97)
Gender (% male)	74.3 *p* = 0.025	64.9 *p* = 0.140	46.2	63.3
Total bacterial DNA (median, range; ng/μL)	5.2 (0.6–27) *p* = 0.249	3.3 (0.5–20) *p* = 0.922	2.6 (0.5–21)	3.3 (0.5–27)
# of *pks*-positive patients	15	19	12	46
*pks* prevalence	43% *p* = 0.798	51% *p* = 0.685	46%	47%

*CRC* colorectal cancer

The *pks* prevalence in each disease category was determined by conventional qualitative PCR. It was 43 and 51% in CRC and adenoma patients, respectively. Neither of them showed significant difference from that in controls, which was 46% (the *p* value was 0.798 and 0.685, respectively) (Table 2). We also calculated *pks* prevalence according to their present endoscopic findings, and no difference was observed either (data not shown).

**Fig. 3:**
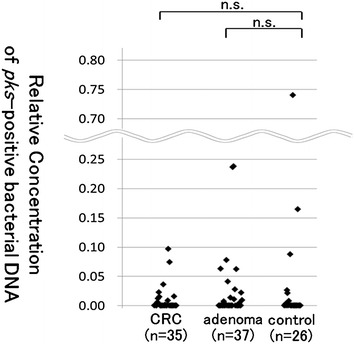
Relative concentrations of *pks*-positive bacterial DNA according to disease status. *CRC* colorectal cancer

Relative concentration of *pks*-positive bacterial DNA in each fecal sample was determined by real-time quantitative PCR. The results are shown in Fig. 3, according to disease category. It ranged from 0 to 0.097 (median 0.00019) in CRC patients, and from 0 to 0.24 (median 0.00025) in adenoma patients. Neither of them showed significant difference from that in controls, which ranged from 0 to 0.74 (median 0.00014) (the *p* value was 0.836 and 0.570, respectively). We also determined relative *pks* concentration according to present endoscopic findings, and there was no significant difference either (data not shown).

**Fig. 4:**
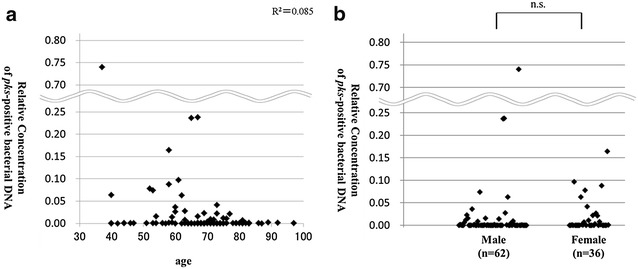
Relative concentrations of *pks*-positive bacterial DNA according to patient age (**a)** and patient gender (**b**)

There was no correlation between patient age and relative *pks* concentration (Fig. 4a). Relative *pks* concentration ranged from 0 to 0.74 (median 0.00018) in male patients and ranged from 0 to 0.16 (median 0.00026) in female patients, which produced no significant difference (Fig. 4b).

### Post-hoc subgroup analysis (CRC patients)

We performed post hoc subgroup analysis on *pks* prevalence in CRC patients for whom some additional information on their CRC was available (Table 3). *pks* prevalence was not significantly different between patients with right-sided CRC (located in cecum, ascending colon, or transverse colon) and those with left-sided CRC (located in descending colon, sigmoid colon, or rectum). We also compared patients whose tumor invading only the mucosa (M) or submucosa (SM), and those with tumor invading the muscularis propria (MP) or further, but no significant difference in *pks* prevalence was detected. Half of the patients with *k*-*ras* mutant CRC were *pks*-positive, while none of those with *k*-*ras* wild-type CRC were *pks*-positive, although this difference in *pks* prevalence did not reach to statistical significance. The presence of previous or ongoing treatment against CRC, or the presence of colonic obstruction due to tumor did not significantly affect *pks* prevalence.

**Table 3:** *pks* prevalence according to CRC subgroups

Subgroup	*pks*-positive	*pks*-negative	Total	*pks* prevalence
Location of CRC				
Right-sided	4	4	8	50%
Left-sided	8	11	19	42%
				*p* = 0.706
Depth of CRC				
M or SM	3	2	5	60%
MP or further	9	13	22	41%
				*p* = 0.438
*k*-*ras* gene status				
Wild-type	0	4	4	0%
Mutant	3	3	6	50%
				*p* = 0.091
Obstruction and treatment status				
Non-obstructed, before treatment	7	5	12	58%
Non-obstructed, under or after treatment	3	8	11	27%
				*p* = 0.133
Obstructed	5	7	12	42%
				*p* = 0.414

*CRC* colorectal cancer, *M* mucosa, *SM* submucosa, *MP* muscularis propria

## Discussion

Our present study is the first to investigate the difference in *pks* prevalence between CRC patients and non-CRC controls in the Japanese population. It is also unique in that it used colonic lavage samples derived during colonoscopy, and the samples were collected and stored in a uniform method performed by a limited number of investigators with good understanding of the study, which might be advantageous compared to asking patients to collect their stool for themselves. Using colonic lavage samples is less invasive compared to biopsy, which also makes this method easy to practice in clinical settings.

This study showed that the prevalence of *pks*-positive *E. coli* was not significantly higher in CRC patients compared to controls. Based on the findings in the previous study [8], we had assumed that *pks* prevalence in CRC and control patients would be 67 and 21%, respectively. According to this assumption and a two-sided α level of 0.05, 35 CRC patients and 26 controls in the present study give the statistical power of 92%, which makes it likely that the result in this study is a true negative.

Using colonic lavage samples, *pks* prevalence in CRC patients was 43%. This value is lower compared to those of CRC patients in two previous studies from Europe using colon tissue specimens, which are 67 and 55%, respectively (Table 4) [8, 9]. On the other hand, *pks* prevalence in our non-CRC controls (46%) appears higher than their counterparts in those studies (21 and 19%, respectively). This discrepancy in *pks* prevalence might simply reflect the difference between mucosa-associated bacteria and those floating in the gut lumen, but in two studies from the United States using stool instead of tissue specimens showed *pks* prevalence of 32 and 20% [14, 20], which are not very different from those using tissue specimens. We also evaluated *pks* prevalence using colonic lavage samples and concurrent biopsy samples from 13 patients, which indicated good correlation. Another possible explanation to this discrepancy is the microbial alteration by colonic lavage procedure before colonoscopy. However, several previous reports show such changes rarely overcome interpersonal differences in bacterial profile at phylum or genus level [21, 22].

**Table 4:** Comparison of *pks* prevalence among previous reports and the present study

	Sample	# of patients	Country	*pks* prevalence
Arthur et al. [8]	Tissue	45	UK	CRC 67% Control 21% (*p* < 0.001)
Buc et al. [9]	Tissue	69	France	CRC 55% Diverticulosis 19% (*p* = 0.0024)
Johnson et al. [14]	Stool (rectal swab)	69	USA	CRC NA Control 32%
Gomez-Moreno et al. [20]	Stool	41	USA (Puerto Rico)	CRC NA Control 20%
Present study	Colonic lavage	61^†^	Japan	CRC 43% Control 46% (*p* = 0.798)

*CRC* colorectal cancer, *NA*​ no answer

^†^ 35 CRC patients and 26 controls

We should also note that healthy Japanese population seems to show relatively high prevalence of phylogenetic group B2 strains among their stool *E. coli* compared to their Western counterparts. In one report from Tokyo, Japan, group B2 strain accounted for 44% of all *E. coli* isolates [23]. On the other hand, this value was 16, 32, 33, and 37% in one report from France [24], 29% in another from the same country [24], and 48% in one from the United States [25]. Taking into account that most *pks*-positive bacteria belong to phylogenetic group B2 *E. coli*, this can lead to high prevalence of *pks*-positive bacteria among healthy Japanese people, including non-CRC controls in this study.

Post-hoc subgroup analysis on CRC patients in this study showed no significant association between clinical parameters and *pks* prevalence. However, it might be worth noting that *pks*-positive *E. coli* was detected only in *k*-*ras* mutant CRC patients, although the difference did not reach to statistical significance due to small sample size. It might imply a possibility that *pks*-positive *E. coli* can induce *k*-*ras* gene mutation through its genotoxic property.

## Conclusions

Although the method of collecting fecal DNA from colonic lavage samples was safe and technically feasible, factors other than *pks*-positive bacteria appear to play more important roles in CRC development in the Japanese population.
